# Supplementary material for: Comparative Analysis of the Complete Chloroplast Genome Sequences of Three Closely Related East-Asian Wild Roses (Rosa sect. Synstylae; Rosaceae)
Source: Genes (Basel). 2019 Jan 3;10(1):23. doi: 10.3390/genes10010023 (PMC6356658; doi:10.3390/genes10010023)
Supplement: Supplementary file 1 [file genes-10-00023-s001.zip › Supplementary Table 1 Gene content list.docx]

**Table S1.** List of genes in chloroplast genomes of three *Rosa* sect. *Synstylae* species

| **Category** | **Group** | **Genes** |
| --- | --- | --- |
| Photosynthesis | Photosystem I protein | *psaA*, *psaB*, *psaC*, *psaI*, *psaJ*, *ycf3***, *ycf4* |
|  | Photosystem II protein | *psbA*, *psbB*, *psbC*, *psbD*, *psbE*, *psbF*, *psbH*, *psbI*, *psbJ*, *psbK*, *psbL*, *psbM*, *psbN*, *psbT*, *psbZ* |
|  | NADH dehydrogenase subunit | *ndhA**, *ndhB***^†^, *ndhC*, *ndhD*, *ndhE*, *ndhF*, *ndhG*, *ndhH*, *ndhI*, *ndhJ*, *ndhK* |
|  | ATP synthase subunit | *atpA*, *atpB*, *atpE*, *atpF*, *atpH*, *atpI* |
|  | Cytochrome complex subunit | *petA*, *petB**, *petD**, *petG*, *petL*, *petN* |
|  | Large subunit of RuBisCO | *rbcL* |
| Ribosomal proteins | Large subunit of ribosome | *rpl2**^†^, *rpl14*, *rpl16**, *rpl20*, *rpl22*, *rpl23*^†^, *rpl32*, *rpl33*, *rpl36* |
|  | Small subunit of ribosome | *rps2*, *rps3*, *rps4*, *rps7*^†^, *rps8*, *rps11*, *rps12**^††^, *rps14*, *rps15*, *rps16**, *rps18*, *rps19* |
| RNA polymerase | DNA-directed RNA polymerase subunit | *rpoA*, *rpoB*, *rpoC1**, *rpoC2* |
| Other proteins | Acetyl-CoA carboxylase subunit | *accD* |
|  | ATP-dependent Clp protease proteolytic subunit | *clpP*** |
|  | Chloroplast envelope membrane protein | *cemA* |
|  | Cytochrome c biogenesis protein | *ccsA* |
|  | Maturase | *matK* |
|  | TIC complex component | (*ycf1*^††^) |
|  | Hypothetical protein | *ycf2*^†^, *orf42*^†^, *orf188* |
| rRNA & tRNA | Ribosomal RNA | *rrn4.5*^†^, *rrn5*^†^, *rrn16*^†^, *rrn23*^†^ |
|  | Transfer RNA | *trnA(UGC)**^†^, *trnC(GCA)*, *trnD(GUC)*, *trnE(UUC)*, *trnF(GAA)*, *trnfM(CAU)*, *trnG(GCC)*, *trnG(UCC)**, *trnH(GUG)*, *trnI(CAU)*^†^, *trnI(GAU)**^†^, *trnK(UUU)**, *trnL(CAA)*^†^, *trnL(UAA)**, *trnL(UAG)*, *trnM(CAU)*, *trnN(GUU)*^†^, *trnP(UGG), trnQ(UUG)*, *trnR(ACG)*^†^, *trnR(UCU)*, *trnS(GCU)*, *trnS(GGA), trnS(UGA), trnT(GGU), trnT(UGU)*, *trnV(GAC)*^†^, *trnV(UAC)**, *trnW(CCA)*, *trnY(GUA)* |

* gene with one intron; ** gene with two introns; ^†^ Gene duplicated to two copies in inverted repeats (IRs); ^††^ Gene partially duplicated in IRs; ( )Tentative gene function
